# Indian clinical research industry

**Published:** 2010

**Authors:** Melanie Fernandes, Arun Bhatt

**Affiliations:** Associate - Medical & Regulatory Affairss; President, Clininvent Research Pvt. Ltd

**Table:** Trials registered in clinicaltrials.gov

	All countries	% Growth	India	% Growth	China	% Growth	Korea	% Growth
2005	10480		137		119		114	
2006	8665	-17.3	203	48.2	157	31.9	205	79.8
2007	9711	12.1	218	7.4	202	28.7	268	30.7
2008	11525	18.7	272	24.8	274	35.6	329	22.8
2009	11173	-3.1	246	-9.6	316	15.3	397	20.7
CAGR %	1.6		15.8		27.7		36.6	

**Table:** Industry trials registered in clinicaltrials.gov

	Industry	% Growth	India	% Growth	China	% Growth	Korea	% Growth
2005	6485		101		78		109	
2006	4707	-27.4	156	54.5	123	57.7	163	49.5
2007	5452	15.8	183	17.3	132	7.3	191	17.2
2008	6707	23.0	220	20.2	146	10.6	241	26.2
2009	6109	-8.9	195	-11.4	107	-26.7	249	3.3
CAGR % 2005-8	1.1%		29.6		23.2		30.3	
CAGR % 2005-9	-1.5		17.9		8.2		22.9	

**Table:** Indian investigators listed in US FDA CDER bioresearch monitoring information system

	No of cities	No of investigators
Cities with >10 sites	20	782
Cities with <10 sites	37	84
Total	57	866

**Table:** City with > 10 investigators

City	No. of Investigators	No. of Institutions	City	No. of Investigators	No. of Institutions
Bengaluru	122	27	Nagpur	20	14
Mumbai	97	32	Cochin	19	4
Hyderabad	94	24	Kolkata	19	13
Chennai	76	25	Coimbatore	18	8
New Delhi	75	24	Ludhiana	18	2
Pune	51	14	Jaipur	17	10
Ahmedabad	38	23	Madurai	13	5
Lucknow	24	3	Mangalore	12	5
Thiruvananthapuram	24	7	Manipal	12	4
Vellore	22	1	Chandigarh	11	1

